# Novel Inhibitor for Downstream Targeting of Transforming Growth Factor-β Signaling to Suppress Epithelial to Mesenchymal Transition and Cell Migration

**DOI:** 10.3390/ijms23095047

**Published:** 2022-05-02

**Authors:** Tsugumasa Toma, Hiroshi Tateishi, Kensaku Kawakami, Taha F. S. Ali, Masahiro Kamo, Kazuaki Monde, Yuta Nakashima, Mikako Fujita, Masami Otsuka

**Affiliations:** 1 Medicinal and Biological Chemistry Science Farm Joint Research Laboratory, Faculty of Life Sciences, Kumamoto University, Kumamoto 862-0973, Japan; 229y2011@st.kumamoto-u.ac.jp (T.T.); taha.ali@mu.edu.eg (T.F.S.A.); sw9317@cc.saga-u.ac.jp (M.K.); motsuka@gpo.kumamoto-u.ac.jp (M.O.); 2 Graduate School of Science and Technology, Kumamoto University, Kumamoto 860-8555, Japan; k.kawakami110@gmail.com; 3 Medicinal Chemistry Department, Faculty of Pharmacy, Minia University, Minia 61519, Egypt; 4 Department of Microbiology, Faculty of Life Sciences, Kumamoto University, Kumamoto 860-8556, Japan; monde@kumamoto-u.ac.jp; 5 Faculty of Advanced Science and Technology, Kumamoto University, Kumamoto 860-8555, Japan; yuta-n@mech.kumamoto-u.ac.jp; 6 International Research Organization for Advanced Science & Technology, Kumamoto University, Kumamoto 860-8555, Japan; 7 Institute of Industrial Nanomaterials, Kumamoto University, Kumamoto 860-8555, Japan; 8 Department of Drug Discovery, Science Farm Ltd., Kumamoto 862-0976, Japan

**Keywords:** anti-cell migration, anti-epithelial to mesenchymal transition (EMT), transforming growth factor-β (TGF-β), non-small-cell lung cancer (NSCLC) cells, Smad

## Abstract

Cancer metastasis accounts for most of the mortality associated with solid tumors. However, antimetastatic drugs are not available on the market. One of the important biological events leading to metastasis is the epithelial to mesenchymal transition (EMT) induced by cytokines, namely transforming growth-factor-β (TGF-β). Although several classes of inhibitors targeting TGF-β and its receptor have been developed, they have shown profound clinical side effects. We focused on our synthetic compound, HPH-15, which has shown anti-fibrotic activity via the blockade of the TGF-β Smad-dependent signaling. In this study, 10 μM of HPH-15 was found to exhibit anti-cell migration and anti-EMT activities in non-small-cell lung cancer (NSCLC) cells. Although higher concentrations are required, the anti-EMT activity of HPH-15 has also been observed in 3D-cultured NSCLC cells. A mechanistic study showed that HPH-15 inhibits downstream TGF-β signaling. This downstream inhibition blocks the expression of cytokines such as TGF-β, leading to the next cycle of Smad-dependent and -independent signaling. HPH-15 has AMPK-activation activity, but a relationship between AMPK activation and anti-EMT/cell migration was not observed. Taken together, HPH-15 may lead to the development of antimetastatic drugs with a new mechanism of action.

## 1. Introduction

Although new and effective therapies are constantly being developed for some types of cancers, cancer remains one of the leading causes of death worldwide [1]. The ability of cancer cells to easily metastasize makes cancer treatment particularly difficult. Cancer metastasis is the migration of cancer cells from the primary tumor to distant locations through blood/lymphatic vessels to form new tumors in remote organs [2,3]. Metastasis accounts for more than 90% of the mortality caused by solid tumors [4,5]. Therefore, the molecular mechanism of metastasis has been extensively studied, and the development of anti-metastatic drugs has been attempted [4,5]. However, such drugs are not on the market yet.

The events of cancer metastasis are complex. One of the important biological events leading to metastasis is the epithelial to mesenchymal transition (EMT) [6,7], in which epithelial cancer cells undergo morphological changes to spindle-like mesenchymal cells with less adhesion between cell-to-cell junctions, ultimately acquiring migratory and invasive capabilities [8]. During EMT, epithelial cells lose their E-cadherin protein localized in the plasma membrane and upregulate the expression of N-cadherin and vimentin. E-cadherin maintains cell adhesion and epithelial structure, while N-cadherin and vimentin increase cell mobility and contribute to the morphological changes of the cell, making them important markers of EMT. EMT is driven by the induction of transcription factors, such as Snail1 and zinc finger E-box binding homeobox 1 (Zeb1) [9,10]. These transcription factors are known to be induced by various cytokines and their downstream signaling depending on the cell type [11].

For example, transforming growth-factor β (TGF-β) is a major inducer of EMT in non-small-cell lung cancer (NSCLC) cells via Smad-dependent and Smad-independent pathways [12,13]. Furthermore, it has been reported that the suppression of these signals inhibits EMT [14,15]. Therefore, TGF-β signaling may be a target of anti-EMT drugs. To date, several classes of inhibitors targeting TGF-β or its receptor have been developed, and some have been clinically tested. Such known agents include TGF-β-neutralizing antibodies, ligand traps that block the interaction between TGF-β and its receptors, and selective small molecules targeting the TGF-β receptor or its kinase inhibitors [16]. One particular example is the low-molecular-weight TGF-β receptor inhibitor, LY3200882, which showed great potential in both in vitro cell models and in vivo animal models [17]. However, as TGF-β has multifaceted functions whose inhibition has led to profound side effects, such inhibitors have not been approved yet [18]. Furthermore, to the best of our knowledge, there are no inhibitors targeting downstream TGF-β signaling.

**Figure 1:**
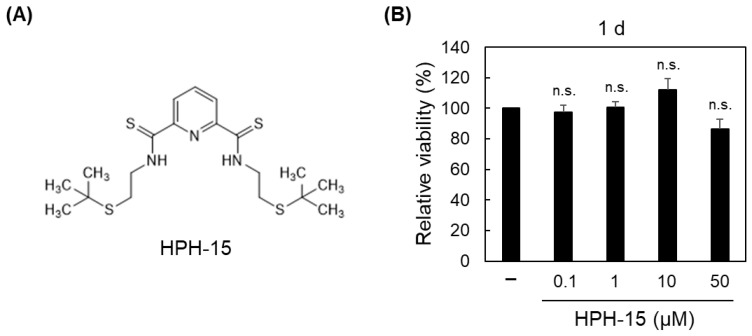

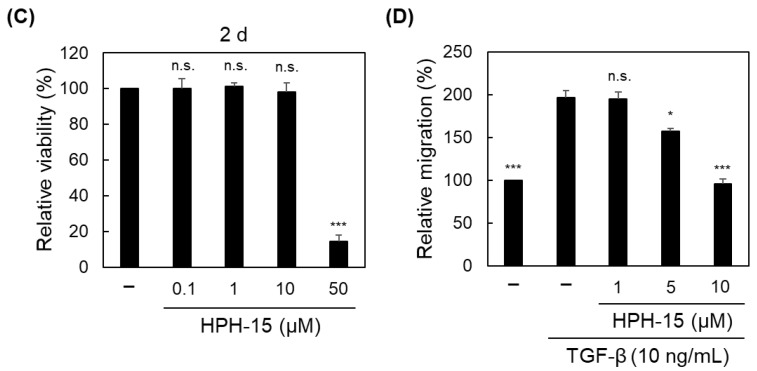
Anti-cell migration activity of HPH-15. (**A**) Structure of HPH-15. (**B**) and (**C**) Viability of A549 cells treated with HPH-15. After incubation of the cells for 1 (**B**) or 2 d (**C**) in the presence of various amounts of HPH-15, MTT assays were performed. Relative values are shown. (**D**) Migration of TGF-β-stimulated A549 cells treated with HPH-15. In vitro scratch assays of the cells, incubated in the presence of various amounts of HPH-15 and TGF-β (10 ng/mL) for 1 d, were performed. Relative values are shown. * *p* < 0.05, *** *p* < 0.001; n.s.—not significant compared with samples without HPH-15 as in (**B**,**C**), and without HPH-15 but with TGF-β as in (D).

We previously reported a low-molecular weight compound, HPH-15 (Figure 1A), which blocked the TGF-β Smad-dependent signaling in dermal fibroblasts and improved skin fibrosis in a mouse model of systemic sclerosis [19]. Here, we report that HPH-15 exerts anti-cell migration and anti-EMT activities in NSCLC cells by downstream targeting of TGF-β signaling, which is a new mechanism as far as we know.

## 2. Results

The anti-migration activity of HPH-15 was examined using a TGF-β-stimulated NSCLC cell line. HPH-15 was synthesized as previously described [20]. The A549 cell line [21], a widely used NSCLC cell line, was used in this study. Before the assay, the toxicity of HPH-15 in the A549 cells was examined. The cells were incubated with 0.1–50 μM of HPH-15 for 1 or 2 d, and the 3-(4,5-dimethylthiazol-2-yl)-2,5-diphenyltetrazolium bromide (MTT) assay was performed. It was demonstrated that 0.1–10 μM of HPH-15 did not show toxicity in both 1 and 2 d incubation but was toxic at 50 μM of HPH-15 in 2 d incubation (Figure 1B,C). Then, 1–10 μM of HPH-15 was used to examine its effect on anti-cell migration activity. The activity was evaluated using an in vitro scratch assay, in which cell migration over 1 d in wound areas of TGF-β-stimulated A549 cells treated with HPH-15 was measured (Figure 1D). In the presence of TGF-β, the cellular area increased more than in the absence of TGF-β due to cell migration. Nearly 40% of this migration was suppressed by 5 μM of HPH-15, and HPH-15 completely inhibited cell migration at 10 μM.

**Figure 2:**
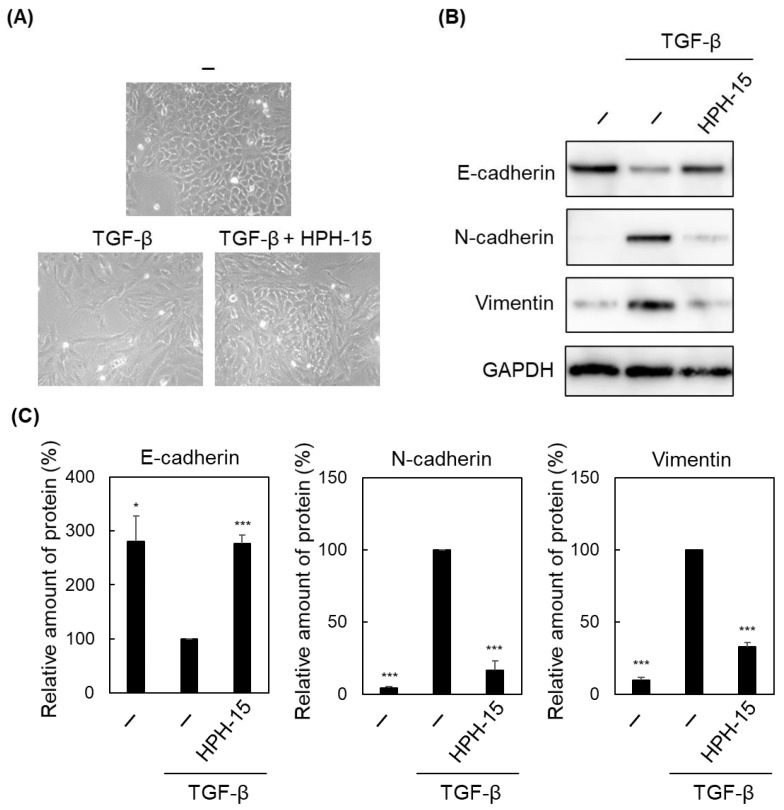

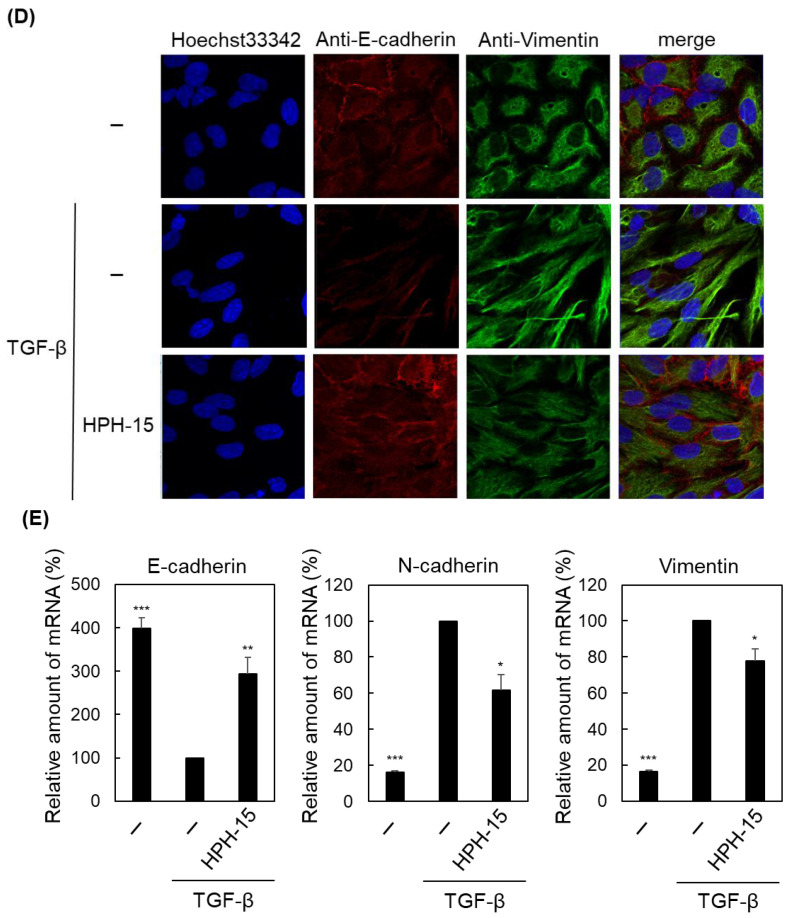
Anti-EMT activity of HPH-15. (**A**) Morphology of TGF-β-stimulated A549 cells with HPH-15. After incubating the cells for 3 d in the presence of HPH-15 (10 μM) and TGF-β (10 ng/mL), cell morphology was observed using a microscope. (**B**) Protein levels of E-cadherin, N-cadherin, and Vimentin in TGF-β-stimulated A549 cells with HPH-15. After incubating the cells for 3 d in the presence of HPH-15 (10 μM) and TGF-β (10 ng/mL), the cells were lysed. The lysate was analyzed by immunoblotting. (**C**) Quantification of the amount of E-cadherin, N-cadherin, and Vimentin. The intensity of the bands in (**B**) was quantified using ImageJ. Each value was normalized by that of GAPDH, and relative values are shown. (**D**) Protein localization of E-cadherin and Vimentin in a TGF-β-stimulated A549 cell treated with HPH-15. After incubating the cells for 3 d in the presence of HPH-15 (10 μM) and TGF-β (10 ng/mL), cellular immunostaining was performed for microscopy. Hoechst33342 was used for nuclear staining. (**E**) mRNA levels of E-cadherin, N-cadherin, and Vimentin in TGF-β-stimulated A549 cells treated with HPH-15. After incubating the cells for 1 d in the presence of HPH-15 (10 μM) and TGF-β (10 ng/mL), RNA was extracted from the cells, and RT-PCR was performed. Relative values are shown. * *p* < 0.05, ** *p* < 0.01, *** *p* < 0.001 compared with samples treated with TGF-β and without HPH-15.

Next, we examined the anti-EMT activity of HPH-15. It has been reported that TGF-β stimulation induces EMT in A549 cells [22]. A549 cells were incubated with TGF-β and HPH-15 (10 μM) for 3 d, and cell morphology was observed under a microscope. A549 cells normally gathered, and there were almost no spaces inside a cell cluster in the absence of TGF-β (Figure 2A). When stimulated with TGF-β, the cell morphology changed to a spindle shape, and spaces were observed between cells. In the presence of both TGF-β and HPH-15, the cell changed the morphology to that similar to normal cells without spaces. A549 cells incubated with both TGF-β and HPH-15 were lysed and immunoblot analysis was performed. It was followed by normalization of total proteins to observe the levels of EMT marker proteins (Figure 2B,C). TGF-β treatment decreased the levels of the epithelial marker E-cadherin and increased those of the mesenchymal markers N-cadherin and vimentin. These changes were suppressed by the HPH-15 treatment. E-cadherin and vimentin protein levels in these cells were also observed by immunostaining. E-cadherin and vimentin were normally localized in the membrane and cytoplasm, respectively (Figure 2D). In the presence of TGF-β and both TGF-β and HPH-15, the same relative amounts of protein as seen in Figure 2B,C were observed. Furthermore, vimentin expression spread in the spindle-shaped area with TGF-β. Next, the mRNA levels of the marker proteins in the cells were examined. After A549 cells were incubated with TGF-β and HPH-15 (10 μM) for 1 d, RNA was extracted, and RT-PCR was performed (Figure 2E). The results showed that TGF-β inhibited the transcription of E-cadherin and enhanced the transcription of N-cadherin and vimentin. Then, HPH-15 suppressed the effects of TGF-β. These results demonstrate that 10 μM of HPH-15 has inhibitory activity against TGF-β-induced EMT.

**Figure 3:**
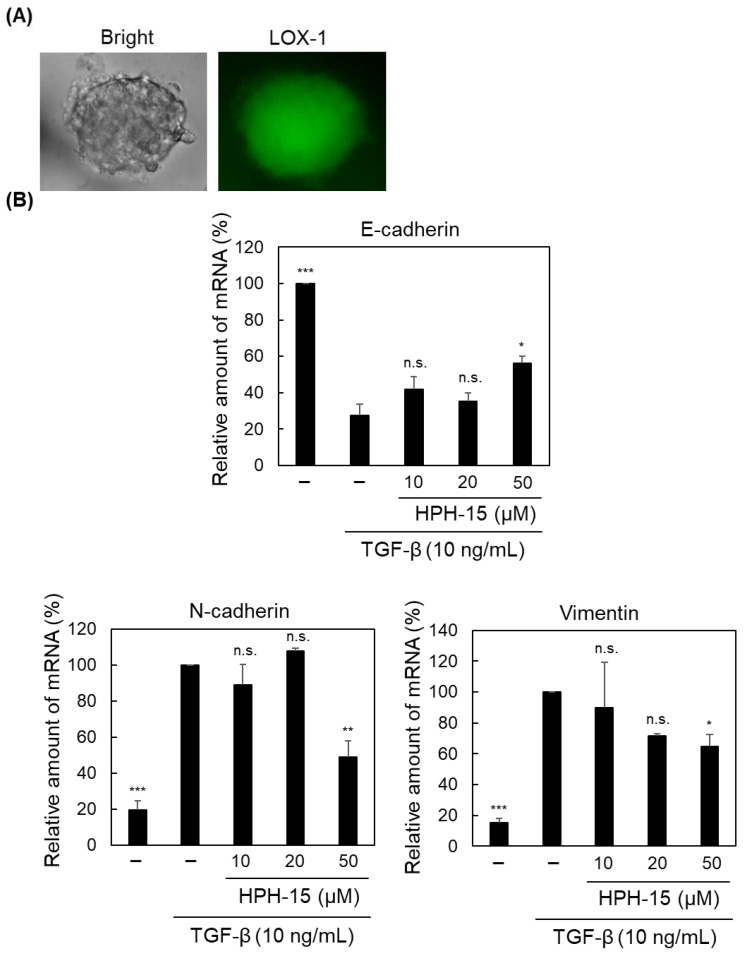
Anti-EMT activity of HPH-15 in 3D-cultured cells. (**A**) Morphology and hypoxic status of a spheroid containing A549 cells. A549 cells were cultured in a non-adhesive plate for 2 d to prepare spheroids, and their morphology was observed using a microscope (left). After incubating the spheroid for 16 h with the hypoxia probe LOX-1, fluorescence microscopy was performed (right). (**B**) mRNA levels of E-cadherin, N-cadherin, and Vimentin in a TGF-β-stimulated A549 spheroid (**A**) treated with HPH-15. After incubating the spheroid for 1 d in the presence of various amounts of HPH-15 and TGF-β (10 ng/mL), RNA was extracted from the spheroid, and RT-PCR was performed. Relative values are shown. * *p* < 0.05, ** *p* < 0.01, *** *p* < 0.001; n.s.—not significant compared with samples treated with TGF-β and without HPH-15.

Recently, three-dimension (3D)-cultured cells have been used in cancer studies because tumors are 3D structures in vivo. Microwells were fabricated, and A549 cells were cultured for 2 d in each microwell. To demonstrate that A549 spheroids formed, the morphology of the cells was observed under a microscope, and their hypoxia status was determined using a chemical probe [23] (Figure 3A). The spheroid was incubated with TGF-β and 10–50 μM of HPH-15 for 1 d. RNA was extracted from the spheroid, and RT-PCR was performed to determine the mRNA levels of EMT marker proteins. As shown in Figure 3B, 10 ng/mL TGF-β suppressed the mRNA level of E-cadherin and increased those of N-cadherin and vimentin, like that observed in Figure 2E. However, in the spheroid, 10 and 20 μM of HPH-15 did not show clear anti-EMT activity, while it was able to inhibit EMT at 50 μM. Notably, 50 μM of HPH-15 was not toxic against normally cultured A549 (Figure 1B) in 1 d incubation. Furthermore, the amounts of mRNA shown in Figure 3B are the value normalized by that of GAPDH. Thus, the effect of HPH-15 at 50 μM is considered to be unrelated to its toxicity. A higher concentration of HPH-15 was required in 3D-cultured cells than in normal 2D-cultured cells.

**Figure 4:**
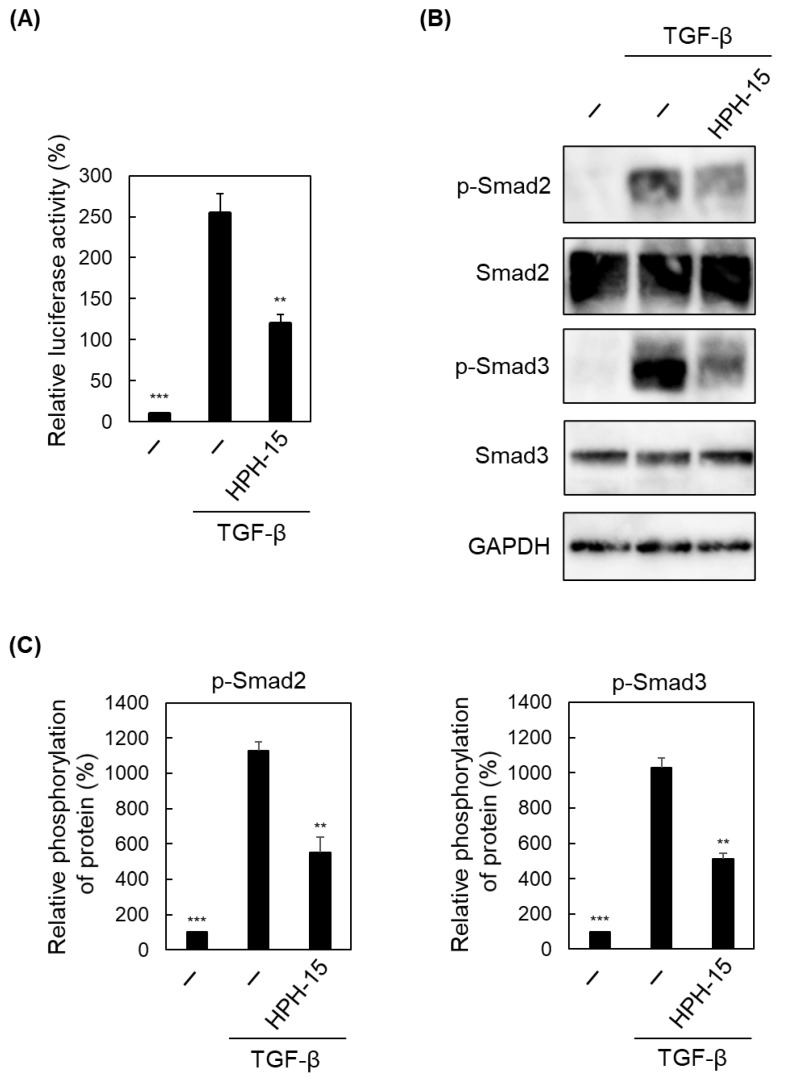

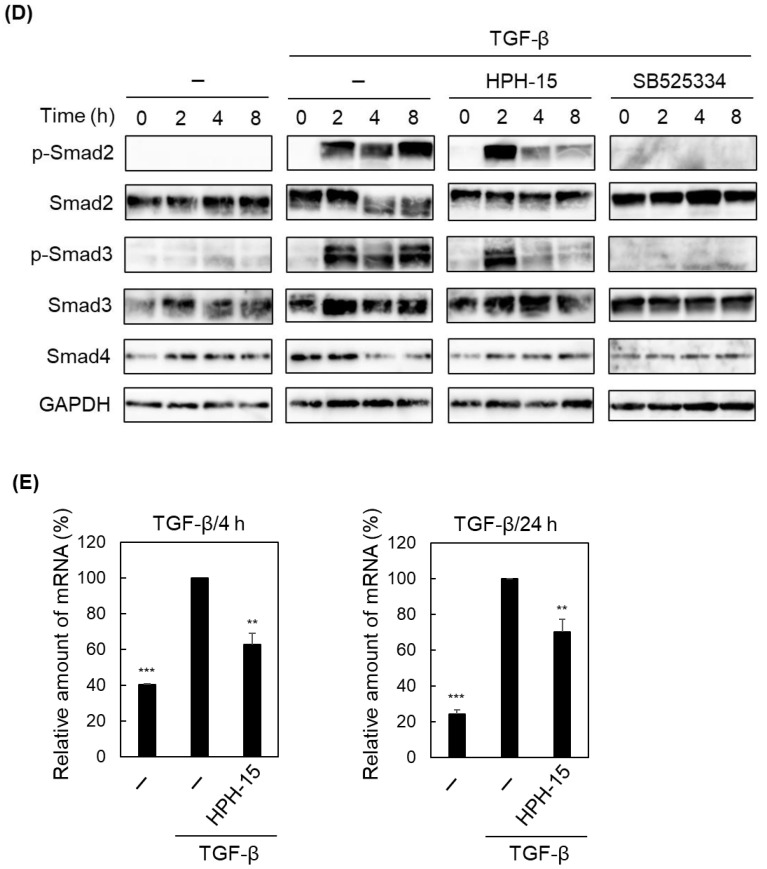
Effects of HPH-15 on a Smad-dependent pathway in TGF-β signaling. (**A**) The activity of a reporter product transcribed from a Smad binding promoter in A549 cells. The cells co-transfected with pGL4.48[*luc2P*/SBE/Hygro] Vector (with SBE and firefly luciferase gene) and pRL-Luc (with a β-actin promoter and renilla luciferase gene for internal control) were incubated for 16 h. After the cells were incubated for another 8 h in the presence of HPH-15 (10 μM) and TGF-β (10 ng/mL), the cells were lysed. The lysate was analyzed by a dual luciferase assay, and the values obtained were normalized. Relative values are shown. (**B**) Protein levels of phosphorylated Smad2 and Smad3 in TGF-β-stimulated A549 cells treated with HPH-15. After incubating the cells for 1 d in the presence of HPH-15 (10 μM) and TGF-β (10 ng/mL), the cells were lysed. The lysate was analyzed by immunoblotting. (**C**) Quantification of phosphorylation of Smad2 and Smad3. The intensity of the bands in (**B**) was quantified using ImageJ. Relative values (band intensity of phosphorylated protein and that of unphosphorylated protein) are shown as values of “Relative phosphorylation of protein.” (**D**) Time course of the amounts of phosphorylated Smad2 and Smad3 proteins in TGF-β-stimulated A549 cells treated with HPH-15. After incubating the cells for the designated time in the presence of HPH-15 (10 μM) or SB525334 (10 μM) and TGF-β (10 ng/mL), the cells were lysed. The lysate was analyzed by immunoblotting. (**E**) mRNA levels of TGF-β1 (TGF-β) in TGF-β-stimulated A549 cells treated with HPH-15. After incubating the cells for 4 or 24 h in the presence of HPH-15 (10 μM) and TGF-β (10 ng/mL), RNA was extracted from the cells, and RT-PCR was performed. Relative values are shown. ** *p* < 0.01, *** *p* < 0.001 compared with samples treated with TGF-β and without HPH-15.

Next, we examined whether HPH-15 exerts its anti-EMT activity via Smad-dependent pathways. First, the transcription from the Smad-binding element (SBE) was examined by a reporter assay using a pGL4.48[*luc2P*/SBE/Hygro] vector (which has SBE fused to a downstream firefly luciferase gene) and pRL-Luc vector (which carries a β-actin promoter fused to a downstream Renilla luciferase gene, which served as an internal control). Cultured A549 cells were co-transfected with these vectors and incubated for 16 h. The cells were then further incubated for 8 h with TGF-β and HPH-15 (10 μM) before a luciferase assay was performed. TGF-β enhanced Smad-dependent transcription, which was inhibited by HPH-15 (Figure 4A). Next, the phosphorylation of Smad2 and Smad3, which plays important roles in Smad-dependent signaling, was examined. After incubation of A549 cells for 1 d with TGF-β and HPH-15 (10 μM), the cells were lysed, and immunoblotting was performed (Figure 4B,C). Without TGF-β, definite phosphorylation of Smad2 and Smad3 was not observed. Upon TGF-β stimulation, both proteins were phosphorylated; upon HPH-15 treatment, the amount of phosphorylated protein was reduced by half. Notably, the levels of Smad2 and Smad3 did not change in the presence of TGF-β or HPH-15. To gain insight into the mechanism of the inhibitory activity of HPH-15, the time course of Smad2/3 phosphorylation was examined. At 2 h post-stimulation with TGF-β, phosphorylation of Smad2 and Smad3 began, and phosphorylation continued up to 8 h (Figure 4D). Smad-dependent signaling is known to express cytokines such as TGF-β that continue to stimulate this signaling, thereby maintaining the transduction of this signaling [24]. Phosphorylation was inhibited throughout this time course in the presence of the TGF-β receptor inhibitor SB525334 [25]. In contrast, while treatment with HPH-15 did not inhibit phosphorylation at 2 h post-stimulation, and inhibition was eventually observed after 4 h. These results suggest that HPH-15 does not directly affect Smad-dependent signaling and inhibits the downstream event of the signaling. In fact, mRNA of TGF-β expressed by TGF-β was suppressed by HPH-15 at 4 and 24 h post-stimulation (Figure 4E).

**Figure 5:**
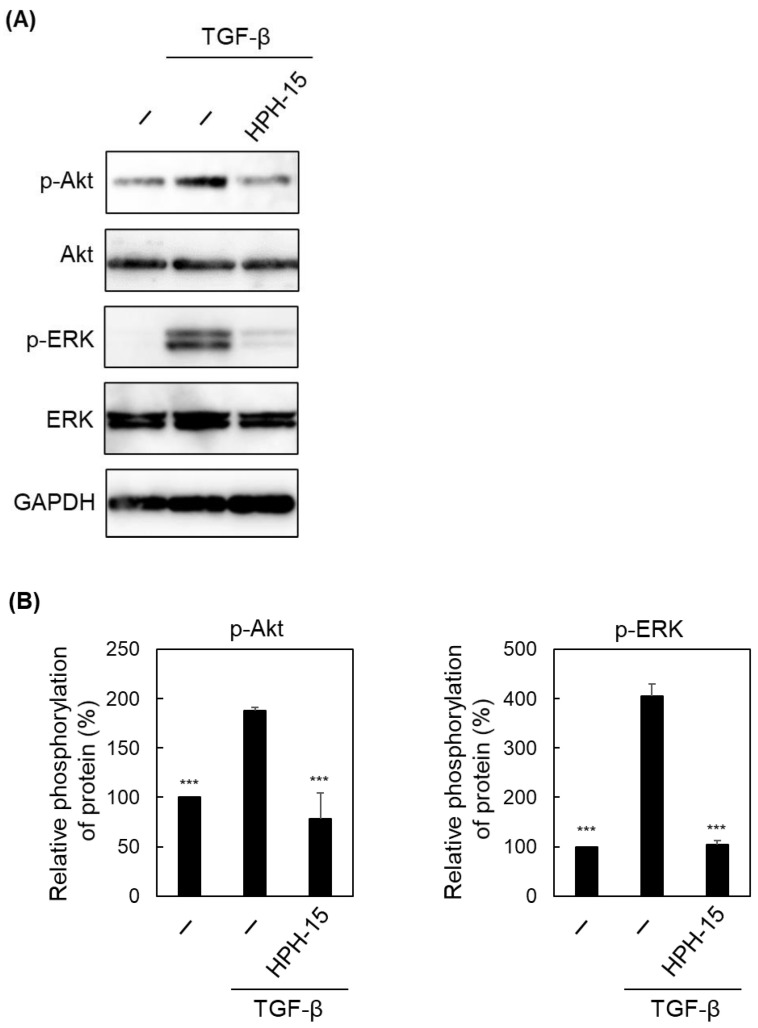
Effects of HPH-15 on a Smad-independent pathway in TGF-β signaling. (**A**) Protein levels of phosphorylated Akt and ERK in TGF-β-stimulated A549 cells treated with HPH-15. After incubating the cells for 1 d in the presence of HPH-15 (10 μM) and TGF-β (10 ng/mL), the cells were lysed. The lysate was analyzed by immunoblotting. (**B**) Quantification of the phosphorylation levels of Akt and ERK. The intensity of the bands in (**A**) was quantified using ImageJ. Relative values (band intensity of phosphorylated protein and that of unphosphorylated protein) are shown as values of “Relative phosphorylation of protein.” *** *p* < 0.001 compared with samples treated with TGF-β and without HPH-15.

Next, we examined the Smad-independent pathway [12,13]. Similar to the experiment to observe the Smad-dependent pathway (Figure 4B), the phosphorylation of Akt and extracellular signal-regulated kinase (ERK) was examined (Figure 5A,B). Upon TGF-β stimulation, phospho-Akt increased, and virtually no phosphorylated ERK was phosphorylated. HPH-15 inhibited the phosphorylation caused by stimulation. The amounts of Akt and ERK did not change with TGF-β/HPH-15 treatment.

**Figure 6:**
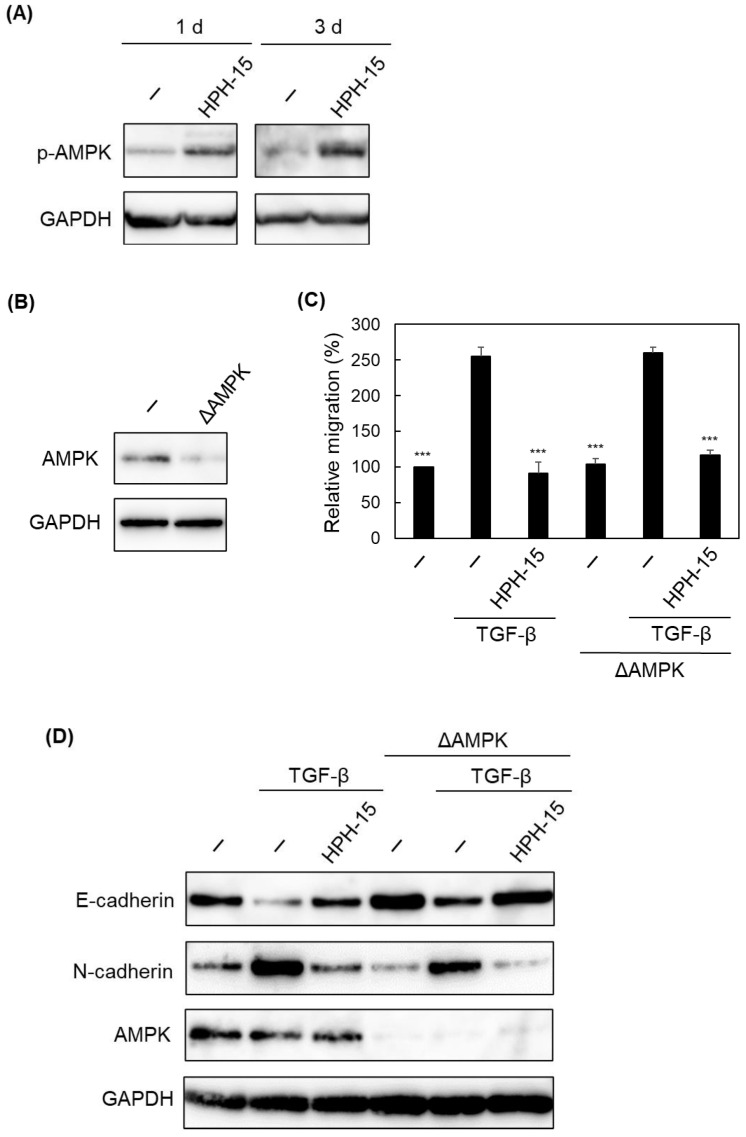
Effects of HPH-15 on AMPK activation. (**A**) Protein levels of phosphorylated AMPK in TGF-β-stimulated A549 cells treated with HPH-15. After incubating the cells for 1 d or 3 d in the presence of HPH-15 (10 μM) and TGF-β (10 ng/mL), the cells were lysed. The lysate was analyzed by immunoblotting. (**B**) Protein levels of AMPK in AMPK-knockdown A549 cells. After transfection of siRNA into A549 cells and incubation for 1 d, the cells were lysed. The lysate was analyzed by immunoblotting. (**C**) Migration of TGF-β-stimulated AMPK-knockdown A549 cells treated with HPH-15. In vitro scratch assays of the cells, incubated with various amounts of HPH-15 and TGF-β (10 ng/mL) for 1 d, were performed. Relative values are shown. (**D**) Protein levels of E-cadherin and N-cadherin in TGF-β-stimulated AMPK-knockdown A549 cells treated with HPH-15. After incubating the cells for 3 d in the presence of HPH-15 (10 μM) and TGF-β (10 ng/mL), the cells were lysed. The lysate was analyzed by immunoblotting. *** *p* < 0.001 compared with samples treated with TGF-β and without HPH-15.

Finally, we focused on the other signaling protein adenosine monophosphate-activated protein kinase (AMPK), since it has been reported that AMPK activation inhibits EMT and cell migration [26]. The effect of HPH-15 on AMPK phosphorylation was examined. A549 cells were incubated for 1 or 3 d with TGF-β and HPH-15 (10 μM) before being lysed to perform immunoblotting (Figure 6A). Interestingly, AMPK was phosphorylated in the presence of HPH-15. AMPK was then knocked down using siRNA (Figure 6B), and the inhibitory activity of HPH-15 on cell migration was examined as described in Figure 1C. Treatment with HPH-15 showed the same anti-cell migration activity as in both A549 and AMPK-knockdown cells (Figure 6C). Furthermore, immunoblot analysis using these two cell lines showed similar anti-EMT activity to those treated with HPH-15 (Figure 6D). These results show that HPH-15 activates AMPK, and the activation is not a cause of its anti-EMT and anti-cell migration properties.

## 3. Discussion

Previously, we reported a compound named SN-1, which has a cysteamine–pyridine–cysteamine structure that binds to the zinc sites of proteins [27,28,29]. Furthermore, we attempted extensive structural modifications by removing and/or introducing substituents [20,30,31,32,33], and achieved improved zinc protein selectivity. HPH-15 is one of them, and we first reported it as an anti-herpes virus compound [20] (note that “HPH-8” in reference [20] was later renamed “HPH-15” when the anti-fibrosis activity of HPH-15 was found [19]). While we classify HPH-15 as one of our metal-binding compounds, the metal binding property seems minimal due to the bulky *tert*-butyl groups. The biological activity of HPH-15 is not directly related to metal binding.

In this study, we focused on the ability of HPH-15 to block the TGF-β Smad-dependent signaling and investigated its anti-cell migration activity. As expected, 10 μM of HPH-15 showed inhibitory activity against TGF-β-driven EMT and cell migration in NSCLC cells. Lung cancer is one of the leading causes of cancer-associated deaths [1], and NSCLC is the most common lung malignancy [34]. Many patients with lung cancer are diagnosed at an advanced stage, and the prognosis of these patients remains very poor owing to early cancer metastasis [34,35]. In many cases, NSCLC metastasizes to the brain to develop brain tumors, which reduces the quality of life of the patients [36].

**Figure 7:**
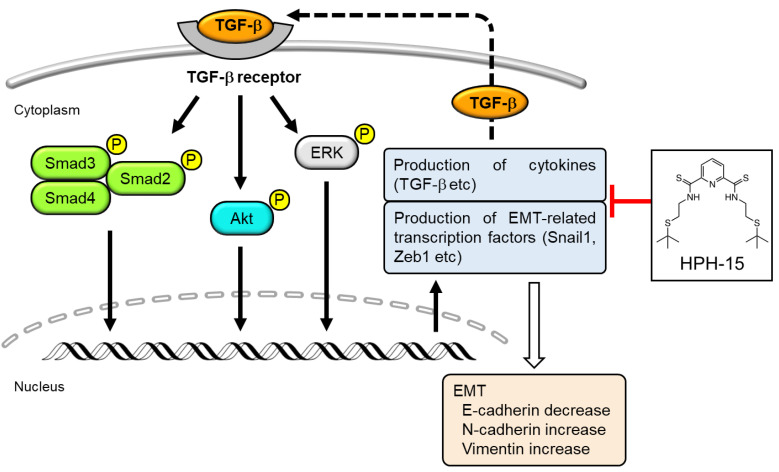
Schematic representation of action mechanism of HPH-15 proposed in this study.

Mechanistic studies showed that HPH-15 inhibited downstream TGF-β signaling (Figure 4D). This downstream inhibition blocks the expression of cytokines such as TGF-β (Figure 4E) that lead to the next cycle of Smad-dependent and Smad-independent signaling. The action mechanism of HPH-15 to inhibit EMT proposed in this study is shown in Figure 7. TGF-β inhibitors are expected to be effective drugs against cancer-related diseases, and various inhibitors targeting TGF-β or its receptor have been developed [16]. However, side effects were observed, and clinical tests failed [18]. The toxicity is caused by the multifaceted functions of TGF-β. As far as TGF-β inhibitors go, the mechanism of inhibition by HPH-15 identified in this study is new, and fewer side effects than those observed from inhibitors targeting the TGF-β receptor are expected.

It is still unclear what the direct target of HPH-15 is and how it leads to the inhibition of TGF-β signaling. Its anti-cell migration and anti-EMT properties were unrelated to AMPK activation activity. While research into the mechanism is ongoing, this study gives us insight into how to develop it into a clinically used anti-metastatic drug. Furthermore, the weaker activity seen in 3D-cultured cells (Figure 3) should be improved. Resistance of 3D-cultured cells to various drugs has been reported [37], and it could be caused by enhancement of drug efflux [38] and/or difficulty of drugs in entering a cell which tightly interacts with adjacent cells. The activity of HPH-15 against 3D-cultured cells may be improved by drug-delivery tactics. This study could lead to the development of new antimetastatic drugs for NSCLC and other cancers.

## 4. Materials and Methods

### 4.1. Chemicals and a Cytokine

HPH-15 was synthesized as previously reported [20] and SB525334 was purchased from Selleck Biotech (Tokyo, Japan). Each compound was dissolved in dimethyl sulfoxide (DMSO) (FUJIFILM-Wako, Osaka, Japan) and the solution was added to the cell-culture medium at a 1:100 volume. TGF-β1 was purchased from R&D Systems (Minneapolis, MN, USA), and used as TGF-β. TGF-β was added to the cell culture medium, followed by the addition of a compound before incubation continued for 1 h.

### 4.2. Cell Culture and Viability

The human NSCLC cell line A549 [21] (provided by the RIKEN BRC through the National Bio-Resource Project of the MEXT/AMED, Japan (RCB0098)) was maintained in Dulbecco’s modified Eagle medium/nutrient mixture F-12 (DMEM/F12) supplemented with 5% heat-inactivated fetal bovine serum (FBS) (Sigma-Aldrich, St. Louis, MO, USA), 89 μg/mL of streptomycin (Meiji Seika Pharma, Tokyo, Japan), and 2.0 μg/mL of amphotericin B (Clinigen, Burton-on-Trent, UK). Cell morphology was observed using a BIOREVO BZ-9000 (Keyence, Osaka, Japan). Cell viability was quantified by the MTT assay, as previously described [39].

### 4.3. Generation of Cancer Spheroids and Hypoxia Assay

The spheroid culture microwells were fabricated by photolithography of SU-8 (KAYAKU Advanced Materials, Westborough, MA, USA) on a coverslip. The device consists of eighty-five microwells with a diameter of 400 μm and a depth of 200 μm. The bottom of each microwell was coated with Prevelex CC1 (Nissan Chemical, Tokyo, Japan). A549 cells (1.0 × 10^6^ cells/mL/well) were seeded in spheroid culture microwells. After incubation for 2 d, an A549 spheroid was formed in each well. The spheroids were incubated with a hypoxia chemical probe LOX-1 (1 μM) (SCIVAX Life Sciences, Tokyo, Japan) for 16 h to analyze their hypoxic status. Fluorescence generated from LOX-1 and spheroid morphology was observed using a BIOREVO BZ-9000 (Keyence).

### 4.4. Protein Knockdown

To knock down AMPK in A549 cells, AMPKα1/2 siRNA (h) (Santa Cruz, Dallas, TX, USA) was transfected into cells using Lipofectamine 3000 (Thermo Fisher Scientific, Waltham, MA, USA). Control siRNA-A (Santa Cruz Biotechnology) was used as a control siRNA. After transfection with siRNA, the cells were incubated for 1 d and used for subsequent experiments.

### 4.5. In Vitro Scratch Assay

The assay was conducted as described previously, in which cell migration for 1 d in wound areas of TGF-β-stimulated A549 cells, prepared by scraping with a 200 μL pipette tip, was measured [40]. The only difference was that an FBS-free medium was used.

### 4.6. Immunostaining of Cells

The assay was conducted as described previously [41]. The differences were as follows: the primary antibodies used were an E-cadherin Antibody (H-108) (Santa Cruz) and a Vimentin Antibody (E-5) (Santa Cruz). The secondary antibodies used were a Goat anti-Mouse IgG (H + L) Cross-Adsorbed Secondary Antibody, Alexa Fluor 488 (Thermo Fisher Scientific), and a Goat anti-Rabbit IgG (H + L) Cross-Adsorbed Secondary Antibody, TRITC (Thermo Fisher Scientific). After the reaction with the secondary antibodies, a phosphate-buffered saline (PBS) solution containing 1% Hoechst33342 (Dojindo Laboratories, Kumamoto, Japan) was added to the cells and incubated for 15 min. A Zeiss LSM 700 laser-scanning confocal microscope (Carl Zeiss, Oberkochen, Germany) was used for fluorescence microscopy.

### 4.7. Immunoblot Analysis

For the analysis of signaling pathways, FBS-free medium was used to culture cells. Cells were lysed using a RIPA buffer (50 mM Tris-HCl (pH 7.8), 150 mM NaCl, 1% Nonidet P-40, 0.5% sodium deoxycholate, 1% protease inhibitor cocktail (Nacalai Tesque, Kyoto, Japan), 1% phosphatase inhibitor cocktail (Nacalai Tesque)), and the protein concentration of the lysate was determined using a BCA Protein Assay Kit (Thermo Fisher Scientific). Gel electrophoresis and immunoblotting were performed using 10 μg total protein per well. As an antibody, E-cadherin Antibody (H-108) (Santa Cruz), N-cadherin Antibody (13A9) (Santa Cruz), Vimentin Antibody (E-5) (Santa Cruz), Phospho-Smad2 (Ser465/467) (138D4) Rabbit mAb (Cell Signaling Technology, Danvers, MA, USA), Smad2 (D43B4) XP Rabbit mAb (Cell Signaling Technology), Phospho-Smad3 (Ser423/425) (C25A9) Rabbit mAb (Cell Signaling Technology), Smad3 (C67H9) Rabbit mAb (Cell Signaling Technology), Smad4 Antibody (B-8) (Santa Cruz), Phospho-Akt (Ser473) (D9E) XP Rabbit mAb (Cell Signaling Technology), Akt (pan) (40D4) Mouse mAb (Cell Signaling Technology), Phospho-SAPK/JNK (Thr183/Tyr185) (81E11) Rabbit mAb (Cell Signaling Technology), ERK1/2 Antibody (MK1) (Santa Cruz), Phospho-AMPKα (Thr172) (40H9) Rabbit mAb (Cell Signaling Technology), AMPKα1/2 Antibody (D-6) (Santa Cruz), or GAPDH (0411) Antibody (Santa Cruz) was used. Immunoreactivity was detected by chemiluminescence using an ImmunoStar LD (FUJIFILM Wako). Band intensity was quantified using the ImageJ software (NIH, Bethesda, MD, USA).

### 4.8. RT-PCR Analysis

The assay was conducted as previously described using the SYBR Green method [40]. Primers for E-cadherin [40], N-cadherin [42], vimentin [43], and TGF-β1 [44] were used as described previously. Glyceraldehyde-3-phosphate dehydrogenase (GAPDH) was analyzed as previously reported [45] and its values were used for normalization.

### 4.9. Luciferase Activity

A549 cells were co-transfected with a pGL4.48[*luc2P*/SBE/Hygro] vector (Promega, Madison, WI, USA) (500 ng) and a pRL-Luc [29] (500 ng) vector using Lipofectamine 3000 (Thermo Fisher Scientific) and incubated for 16 h. HPH-15 was then added and allowed to continue to incubate for 1 h before TGF-β was added. The cells continued to incubate for 8 h. The lysate was analyzed using the Dual-Luciferase Reporter Assay System (Promega). Firefly luciferase (transcribed from pGL4.48[*luc2P*/SBE/Hygro] Vector) activity was normalized with Renilla luciferase (transcribed from pRL-Luc) activity.

## 5. Conclusions

A small molecule, HPH-15, was found to have anti-EMT and anti-cell-migration properties in NSCLC cells. To the best of our knowledge, the mechanism of inhibition downstream of TGF-β signaling is new, although inhibitors targeting the TGF-β receptor or its kinase are known. The elucidation of mechanistic details is ongoing. Cancer metastasis is the main cause of mortality in solid tumors, and anti-metastatic drugs are not yet available. This study could lead to the development of anti-metastasis drugs in the near future.

## Data Availability

Not applicable.

## References

[1] Sung H., Ferlay J., Siegel R.L., Laversanne M., Soerjomataram I., Jemal A., Bray F. (2021). Global Cancer Statistics 2020: GLOBOCAN Estimates of Incidence and Mortality Worldwide for 36 Cancers in 185 Countries. CA Cancer J. Clin..

[2] Gupta G.P., Massagué J. (2006). Cancer metastasis: Building a framework. Cell.

[3] Chaffer C.L., Weinberg R.A. (2011). A perspective on cancer cell metastasis. Science.

[4] Gandalovičová A., Rosel D., Fernandes M., Veselý P., Heneberg P., Čermák V., Petruželka L., Kumar S., Sanz-Moreno V., Brábek J. (2017). Migrastatics-Anti-metastatic and Anti-invasion Drugs: Promises and Challenges. Trends Cancer.

[5] Anderson R.L., Balasas T., Callaghan J., Coombes R.C., Evans J., Hall J.A., Kinrade S., Jones D., Jones P.S., Jones R. (2019). A framework for the development of effective anti-metastatic agents. Nat. Rev. Clin. Oncol..

[6] Heerboth S., Housman G., Leary M., Longacre M., Byler S., Lapinska K., Willbanks A., Sarkar S. (2015). EMT and tumor metastasis. Clin. Transl. Med..

[7] Karlsson M.C., Gonzalez S.F., Welin J., Fuxe J. (2017). Epithelial-mesenchymal transition in cancer metastasis through the lymphatic system. Mol. Oncol..

[8] Le Bras G.F., Taubenslag K.J., Andl C.D. (2012). The regulation of cell-cell adhesion during epithelial-mesenchymal transition, motility and tumor progression. Cell Adh. Migr..

[9] Gheldof A., Berx G. (2013). Cadherins and epithelial-to-mesenchymal transition. Prog. Mol. Biol. Transl. Sci..

[10] Mendez M.G., Kojima S., Goldman R.D. (2010). Vimentin induces changes in cell shape, motility, and adhesion during the epithelial to mesenchymal transition. FASEB J..

[11] Ribatti D., Tamma R., Annese T. (2020). Epithelial-Mesenchymal Transition in Cancer: A Historical Overview. Transl. Oncol..

[12] Derynck R., Zhang Y.E. (2003). Smad-dependent and Smad-independent pathways in TGF-beta family signalling. Nature.

[13] Bierie B., Moses H.L. (2006). Tumour microenvironment: TGFbeta: The molecular Jekyll and Hyde of cancer. Nat. Rev. Cancer.

[14] Yang H., Zhan L., Yang T., Wang L., Li C., Zhao J., Lei Z., Li X., Zhang H.T. (2015). Ski prevents TGF-β-induced EMT and cell invasion by repressing SMAD-dependent signaling in non-small cell lung cancer. Oncol. Rep..

[15] Lee J.H., Chinnathambi A., Alharbi S.A., Shair O.H.M., Sethi G., Ahn K.S. (2019). Farnesol abrogates epithelial to mesenchymal transition process through regulating Akt/mTOR pathway. Pharmacol. Res..

[16] Ciardiello D., Elez E., Tabernero J., Seoane J. (2020). Clinical development of therapies targeting TGFβ: Current knowledge and future perspectives. Ann. Oncol..

[17] Xu G., Zhang Y., Wang H., Guo Z., Wang X., Li X., Chang S., Sun T., Yu Z., Xu T. (2020). Synthesis and biological evaluation of 4-(pyridin-4-oxy)-3-(3,3-difluorocyclobutyl)-pyrazole derivatives as novel potent transforming growth factor-β type 1 receptor inhibitors. Eur. J. Med. Chem..

[18] Teixeira A.F., Ten Dijke P., Zhu H.J. (2020). On-Target Anti-TGF-β Therapies Are Not Succeeding in Clinical Cancer Treatments: What Are Remaining Challenges?. Front. Cell Dev. Biol..

[19] Luong V.H., Chino T., Oyama N., Matsushita T., Sasaki Y., Ogura D., Niwa S.I., Biswas T., Hamasaki A., Fujita M. (2018). Blockade of TGF-β/Smad signaling by the small compound HPH-15 ameliorates experimental skin fibrosis. Arthritis Res. Ther..

[20] Hosono T., Yokomizo K., Hamasaki A., Okamoto Y., Okawara T., Otsuka M., Mukai R., Suzuki K. (2008). Antiviral activities against herpes simplex virus type 1 by HPH derivatives and their structure-activity relationships. Bioorg. Med. Chem. Lett..

[21] Giard D.J., Aaronson S.A., Todaro G.J., Arnstein P., Kersey J.H., Dosik H., Parks W.P. (1973). In vitro cultivation of human tumors: Establishment of cell lines derived from a series of solid tumors. J. Natl. Cancer Inst..

[22] Karacosta L.G., Anchang B., Ignatiadis N., Kimmey S.C., Benson J.A., Shrager J.B., Tibshirani R., Bendall S.C., Plevritis S.K. (2019). Mapping lung cancer epithelial-mesenchymal transition states and trajectories with single-cell resolution. Nat. Commun..

[23] Zhang S., Hosaka M., Yoshihara T., Negishi K., Iida Y., Tobita S., Takeuchi T. (2010). Phosphorescent light-emitting iridium complexes serve as a hypoxia-sensing probe for tumor imaging in living animals. Cancer Res..

[24] Kashiwagi I., Morita R., Schichita T., Komai K., Saeki K., Matsumoto M., Takeda K., Nomura M., Hayashi A., Kanai T. (2015). Smad2 and Smad3 Inversely Regulate TGF-β Autoinduction in Clostridium butyricum-Activated Dendritic Cells. Immunity.

[25] Grygielko E.T., Martin W.M., Tweed C., Thornton P., Harling J., Brooks D.P., Laping N.J. (2005). Inhibition of gene markers of fibrosis with a novel inhibitor of transforming growth factor-beta type I receptor kinase in puromycin-induced nephritis. J. Pharmacol. Exp. Ther..

[26] Lin H., Li N., He H., Ying Y., Sunkara S., Luo L., Lv N., Huang D., Luo Z. (2015). AMPK Inhibits the Stimulatory Effects of TGF-β on Smad2/3 Activity, Cell Migration, and Epithelial-to-Mesenchymal Transition. Mol. Pharmacol..

[27] Fujita M., Otsuka M., Sugiura Y. (1996). Metal-chelating inhibitors of a zinc finger protein HIV-EP1. Remarkable potentiation of inhibitory activity by introduction of SH groups. J. Med. Chem..

[28] Ejima T., Hirota M., Mizukami T., Otsuka M., Fujita M. (2011). An anti-HIV-1 compound that increases steady-state expression of apoplipoprotein B mRNA-editing enzyme-catalytic polypeptide-like 3G. Int. J. Mol. Med..

[29] Koga R., Radwan M.O., Ejima T., Kanemaru Y., Tateishi H., Ali T.F.S., Ciftci H.I., Shibata Y., Taguchi Y., Inoue J.I. (2017). A Dithiol Compound Binds to the Zinc Finger Protein TRAF6 and Suppresses Its Ubiquitination. ChemMedChem.

[30] Hamasaki A., Naka H., Tamanoi F., Umezawa K., Otsuka M. (2003). A novel metal-chelating inhibitor of protein farnesyltransferase. Bioorg. Med. Chem. Lett..

[31] Tanaka A., Radwan M.O., Hamasaki A., Ejima A., Obata E., Koga R., Tateishi H., Okamoto Y., Fujita M., Nakao M. (2017). A novel inhibitor of farnesyltransferase with a zinc site recognition moiety and a farnesyl group. Bioorg. Med. Chem. Lett..

[32] Radwan M.O., Koga R., Hida T., Ejima T., Kanemaru Y., Tateishi H., Okamoto Y., Inoue J.I., Fujita M., Otsuka M. (2019). Minimum structural requirements for inhibitors of the zinc finger protein TRAF6. Bioorg. Med. Chem. Lett..

[33] Tateishi H., Tateishi M., Radwan M.O., Masunaga T., Kawatashiro K., Oba Y., Oyama M., Inoue-Kitahashi N., Fujita M., Okamoto Y. (2021). A New Inhibitor of ADAM17 Composed of a Zinc-Binding Dithiol Moiety and a Specificity Pocket-Binding Appendage. Chem. Pharm. Bull. (Tokyo).

[34] Alexander M., Kim S.Y., Cheng H. (2020). Update 2020: Management of Non-Small Cell Lung Cancer. Lung.

[35] Niu F.Y., Zhou Q., Yang J.J., Zhong W.Z., Chen Z.H., Deng W., He Y.Y., Chen H.J., Zeng Z., Ke E.E. (2016). Distribution and prognosis of uncommon metastases from non-small cell lung cancer. BMC Cancer.

[36] Mujoomdar A., Austin J.H., Malhotra R., Powell C.A., Pearson G.D., Shiau M.C., Raftopoulos H. (2007). Clinical predictors of metastatic disease to the brain from non-small cell lung carcinoma: Primary tumor size, cell type, and lymph node metastases. Radiology.

[37] Fontoura J.C., Viezzer C., Dos Santos F.G., Ligabue R.A., Weinlich R., Puga R.D., Antonow D., Severino P., Bonorino C. (2020). Comparison of 2D and 3D cell culture models for cell growth, gene expression and drug resistance. Mater. Sci. Eng. C Mater. Biol. Appl..

[38] Oshikata A., Matsushita T., Ueoka R. (2011). Enhancement of drug efflux activity via MDR1 protein by spheroid culture of human hepatic cancer cells. J. Biosci. Bioeng..

[39] Ciftci H.I., Ozturk S.E., Ali T.F.S., Radwan M.O., Tateishi H., Koga R., Ocak Z., Can M., Otsuka M., Fujita M. (2018). The First Pentacyclic Triterpenoid Gypsogenin Derivative Exhibiting Anti-ABL1 Kinase and Anti-chronic Myelogenous Leukemia Activities. Biol. Pharm. Bull..

[40] Kamo M., Ito M., Toma T., Gotoh H., Shimozono R., Nakagawa R., Koga R., Monde K., Tateishi H., Misumi S. (2021). Discovery of anti-cell migration activity of an anti-HIV heterocyclic compound by identification of its binding protein hnRNP M. Bioorg. Chem..

[41] Yamamoto M., Koga R., Fujino H., Shimagaki K., Ciftci H.I., Kamo M., Tateishi H., Otsuka M., Fujita M. (2017). Zinc-binding site of human immunodeficiency virus 2 Vpx prevents instability and dysfunction of the protein. J. Gen. Virol..

[42] Basu M., Bhattacharya R., Ray U., Mukhopadhyay S., Chatterjee U., Roy S.S. (2015). Invasion of ovarian cancer cells is induced byPITX2-mediated activation of TGF-β and Activin-A. Mol. Cancer.

[43] Bae D.S., Handa R.J., Yang R.S., Campain J.A. (2003). Gene expression patterns as potential molecular biomarkers for malignant transformation in human keratinocytes treated with MNNG, arsenic, or a metal mixture. Toxicol. Sci..

[44] Gomes L.R., Terra L.F., Wailemann R.A., Labriola L., Sogayar M.C. (2012). TGF-β1 modulates the homeostasis between MMPs and MMP inhibitors through p38 MAPK and ERK1/2 in highly invasive breast cancer cells. BMC Cancer.

[45] Valente V., Teixeira S.A., Neder L., Okamoto O.K., Oba-Shinjo S.M., Marie S.K., Scrideli C.A., Paçó-Larson M.L., Carlotti C.G. (2009). Selection of suitable housekeeping genes for expression analysis in glioblastoma using quantitative RT-PCR. BMC Mol. Biol..

